# A universal pipeline MosaicProt enables large-scale modeling and detection of chimeric protein sequences for studies on programmed ribosomal frameshifting

**DOI:** 10.1016/j.csbj.2025.11.023

**Published:** 2025-11-12

**Authors:** Umut Çakır, Noujoud Gabed, Ali Yurtseven, Igor Kryvoruchko

**Affiliations:** a Clinical Neuroscience Research Group, Max Planck Institute for Multidisciplinary Sciences, The University of Göttingen, Göttingen 37075, Germany; b Cellular and Molecular Biology Department, Oran High School of Biological Sciences (ESSBO), Oran 31000, Algeria; c Faculty of Electrical and Electronics Engineering, Istanbul Technical University, Istanbul 34485, Turkey; d Department of Biology, United Arab Emirates University, P.O. Box 15551, Al Ain, United Arab Emirates

**Keywords:** Chimeric peptide, Programmed ribosomal frameshifting, Alternative open reading frame, Proteome, Mass spectrometry proteomics, Mosaic translation

## Abstract

Peptides and proteins produced by programmed ribosomal frameshifting (PRF) are well-known in viruses. In non-viral systems, only a few examples of such chimeric sequences have been documented until recently. Three studies in eukaryotes showed that chimeric peptides are numerous and diverse. In ciliates, such peptides are associated with stop codons. In humans, their discovery was possible due to focusing on sequences with naturally repeated codons. This way, many candidate sequences with mass spectrometry (MS) proteomics-based support for translation have been identified. In a plant study, our group discovered MS-validated chimeric peptides using a unique modeling algorithm, MosaicProt, which is described and made available here. Our pipeline enables the identification of chimeric peptides in any organism for which transcript sequences and MS proteomic data are available. By design, our approach does not require prior knowledge about sequence similarity to already characterized PRF sites and can detect forward and backward frameshifts by 1 and 2 nucleotides. Thus, our pipeline opens a path for uncovering previously unknown PRF events across various transcript types, potentially broadening our understanding of proteome diversity. The pipeline was designed primarily for studies on mosaic translation, hence the name MosaicProt. However, it is applicable for research on PRF in many different contexts.

## Graphical Abstract

**Figure:**
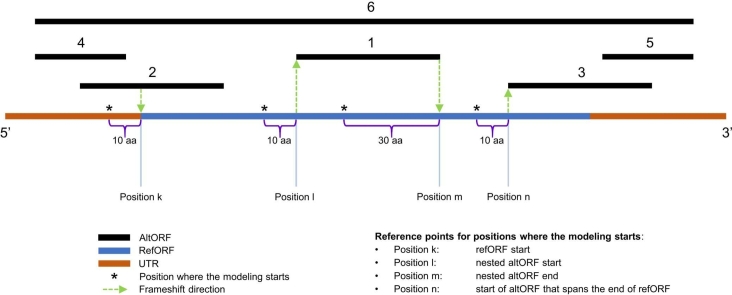

## 1 Introduction

This article is part of a series that presents two novel tools and their validation for research on programmed ribosomal frameshifting (PRF). The first tool, MosaicProt, which is described here, offers a straightforward yet powerful mass spectrometry (MS)-based method for detecting PRF events in any system. The second tool, ChiMSource, efficiently addresses one fundamental challenge in studies on non-canonical translation events, including translation of chimeric proteins via PRF [1]. It predicts all genomic origins of chimeric and non-chimeric MS peptides detected in a biological sample, thus discriminating between MS peptides with unique origin and peptides that can potentially have multiple origins. The functionality of ChiMSource is unique and not implemented in any other pipeline. The third article demonstrates the value of MosaicProt and ChiMSource in searches for previously unknown PRF events [2]. It describes the discovery of 156 chimeric peptides in the legume model plant *Medicago truncatula* and provides evidence for the biological relevance of the dataset.

Before 1985, it was assumed that PRF and the resulting chimeric proteins were phenomena unique to viruses. Two studies conducted in *Escherichia coli* were probably the first to suggest that PRF can have a biological role in a non-viral system [3], [4]. By 2006, it became clear that PRF is found in all kingdoms of life [5]. So far only a few genes have been convincingly shown to use PRF for various purposes in prokaryotes [3], [4], [6], [7], [8], [9], [10] and eukaryotes [11], [12], [13], [14], [15]. The pioneering study in ciliates of genus *Euplotes* was the first to detect multiple chimeric peptides in a non-viral system [13]. All 13 MS-validated chimeric peptides in these organisms originate from sites where a ribosome shifts to an alternative frame at an in-frame stop codon. This PRF activity bypasses stop codons, thus effectively extending the length of translation products. Based on earlier bioinformatic predictions, it was proposed that up to 10 % of human genes and genes in all eukaryotic genomes can undergo PRF [17], [18], [16]. However, experimental evidence for a high abundance of chimeric peptides in humans was obtained only in 2024. Ren and associates found 405 unique MS-supported chimeric peptides in 32 normal human samples [15]. These peptides are thought to originate from 454 loci which have naturally occurring repeat codon sequences at the putative PRF sites. Functional characterization of one such locus, which encodes a histone deacetylase HsHDAC1, revealed that the frameshifted product inhibits the non-frameshifted version of HsHDAC1 [15]. Our parallel study in the model plant *M. truncatula* [2] is conceptually different from the work of Ren et al. [15]. Although it lacks functional validation, we obtained MS support for the translation of 156 chimeric peptides that are not limited to products of mRNA and PRF sites with repeated codons. In addition to mRNA-derived chimeric peptides, we also found that some ncRNA, rRNA, and even tRNA transcripts could potentially produce chimeric peptides. Furthermore, in contrast to the human study, which was focused on PRF events with values −1 and + 1, we extended our scope to include “long” PRF events, encompassing −2 and + 2 frameshifts. Multiple statistically significant observations summarized in Supplementary Datasets S29 and S30 of our data article [2] strongly suggest that the 156 chimeric peptides are biologically relevant and are not a collection of false positives. The discovery of these diverse chimeric sequences in the plant proteome was possible due to our original approach to identifying chimeric peptides via MS, as proposed in our viewpoint article introducing the mosaic translation hypothesis [19]. It is based on the identification of conserved and/or translated alternative open reading frames (altORFs) and using them as “building blocks” for chimeric protein models when altORFs overlap with each other and/or with the main annotated ORF (refORF). Translated altORFs and their products, altProts, are thought to be ubiquitous in all organisms, and many of them have been implicated in vital cellular functions [20], [21], [22]. A recently developed database, OpenProt, provides a comprehensive inventory of altORFs across multiple species [25], [23], [24].

How does MosaicProt stand relative to traditional methods of PRF detection? There are three groups of methods capable of such detection: (1) similarity-based predictions [26], [27], [28], [29]; (2) ribosome profiling, or Ribo-Seq [30], [31], [32], [33]; and (3) MS proteomics-based methods [15], [34]. Similarity-based methods can be useful guides in searches for biologically relevant PRF events. However, the conceptual weakness of such methods is evident from the low evolutionary conservation of most PRF sites, even within the same species. Furthermore, similarity-based prediction methods cannot detect completely novel PRF sites. Often prediction algorithms focus on one or two types of PRF or a specific group of organisms. Ribosome profiling, or Ribo-Seq has been instrumental for the detection of the first large number of chimeric peptides in eukaryotes [13]. However, Ribo-Seq-derived evidence for a frameshift position is indirect compared to MS proteomics-based evidence. Ribo-Seq data are costly and technically challenging to generate, and ribosome association does not necessarily imply active translation. Moreover, using Ribo-Seq for the detection of translation in different frames that overlap is conceptually prone to misinterpretation, as was emphasized by Álvarez-Urdiola and Riechmann [20]. Additionally, many organisms have no Ribo-Seq datasets deposited publicly, including the model plant *M. truncatula*. While MosaicProt does not rely upon the detection of PRF sites with Ribo-Seq, it can benefit from Ribo-Seq data that indicate translation of individual altORFs, for example, data from OpenProt. Like all methods, MS proteomics-based protocols for PRF detection have their own limitations such as potentially high rate of false discoveries. However, they provide the most direct evidence for PRF events and their possible positions. Finding the exact position of a PRF site based on the sequence of a chimeric MS peptide may be challenging because such peptides are usually short and can potentially originate from multiple genomic locations. We addressed this challenge by developing ChiMSource, a pipeline that discriminates between PRF products with unique and multiple sources [1]. MosaicProt implements a prediction- and Ribo-Seq-independent method by generating a very large number of unbiased chimeric protein models for MS-based validation. It does not analyze MS spectra on its own, but it makes it possible to validate previously unsuspected PRF events based on the analysis of MS spectra with the best tools developed for this task, such as the combination of SearchGUI [35] and PeptideShaker [36].

Our original pipeline described in this publication was implemented in *M. truncatula*. However, it can be applied to any organism for which transcriptome and MS proteomic data are available. By enabling systematic detection of PRF-derived peptides, this pipeline has the potential to uncover previously unrecognized aspects of proteome diversity and reveal novel biological functions associated with PRF.

## 2 Material and methods

**Fig. 1:**
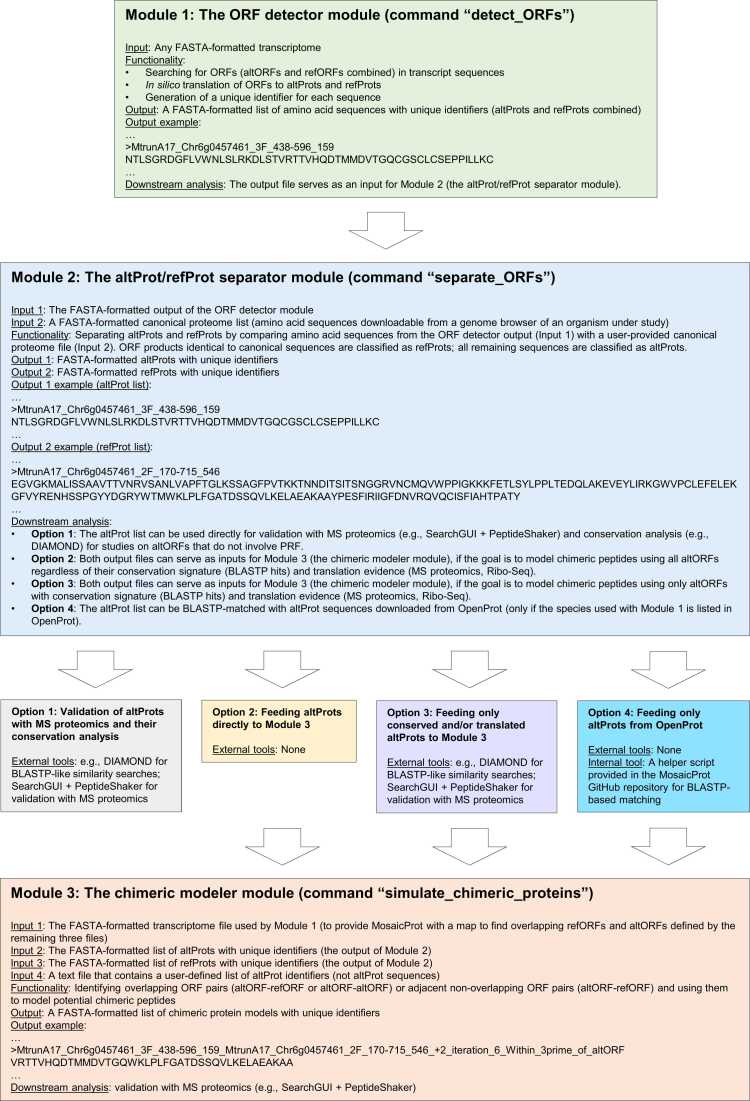
A workflow of MosaicProt, which is the detection pipeline for the mass spectrometry (MS)-based discovery of chimeric peptides. MosaicProt consists of three modules that can be executed sequentially or stopped at different stages depending on the purpose. In Option 1, the workflow ends after altProt validation with MS proteomics, without proceeding to the chimeric modeling step (Module 3). Option 2 involves no intermediate external tools, so that altProts are fed directly to Module 3, without evidence for conservation and/or translation. This can help find the MS support for chimeric peptides generated from taxonomically restricted ORFs. Option 3 helps focus the modeling of chimeric proteins only on altProts with evidence for conservation and/or translation. Option 4 is suitable for modeling with altProts that are conserved and/or translated according to OpenProt.

**Fig. 2:**
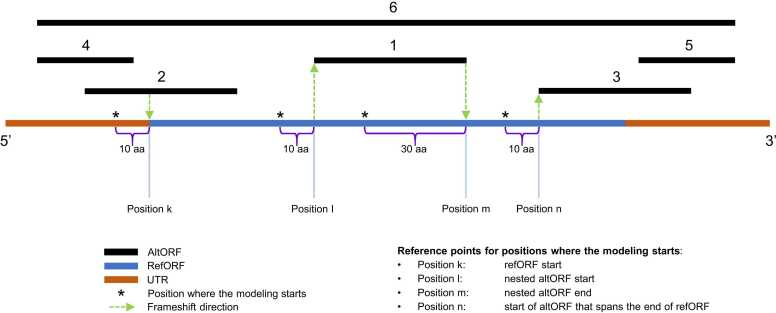
A graphical summary of scenarios handled by the chimeric modeler module. AltORFs and a refORF are shown with black and blue horizontal lines, respectively, and the UTRs are depicted in pale brown. AltORFs can be located in six different positions (black numbers above the lines) relative to their refORF, which requires consideration of distinct scenarios. Scenario 1: altORF 1 is embedded in the refORF. Scenario 2: altORF 2 (or altORF 3) overlaps with the refORF. Scenario 3: altORF 4 (or altORF 5) is located entirely in the UTR and does not overlap with the refORF. The shifts from altORF 6 to the refORF (the 5’-side) and from the refORF to altORF 6 (the 3’-side) are considered as special cases of Scenario 2. Green arrows indicate the frameshift direction in all models corresponding to a specific scenario. For example, all left-side models in Scenario 1 simulate a frameshift from the refORF to the altORF.

In this manuscript, we use the term “chimeric peptide” for short amino acid sequences deduced from MS proteomics, representing segments produced through PRF. For *in silico* generated models, we use the term "chimeric protein model". They are hypothetical constructs that can be matched to MS-derived chimeric peptides and may represent fragments of longer chimeric or mosaic proteins. When discussing these sequences without specifying their lengths, we refer to them broadly as "chimeric proteins". MosaicProt is a streamlined pipeline without built-in modules for conservation analyses, Ribo-Seq, or MS searches. Instead, it addresses a task not tackled by any other pipeline so far: large-scale modeling of PRF products for subsequent MS-validation. Our pipeline consists of three modules that correspond to different steps in the preparation of chimeric protein models: (1) the detection and *in silico* translation of ORFs, (2) the removal of refProts, and (3) the modeling of chimeric proteins based on altProts (Fig. 1). The first module identifies putative ORFs within transcript sequences and performs *in silico* translation to generate potential protein sequences. Outputs of this module include both altProts and refProts. In the second module, refProts are removed from the list of potential protein sequences deduced by the first module. Its function is to disable the MS detection of signals that come from refProts, thus focusing on non-canonical sequences. Module 2 outputs can be used directly as inputs for the third module regardless of evidence for conservation or translation of altProts (see below). The third module generates chimeric protein models based on user-defined criteria, e.g., a list of conserved, Ribo-Seq-supported, and/or MS-validated altProts. The user may choose to obtain evidence for conservation and translation independently, like we did in our study. Alternatively, MosaicProt can be applied to species listed in OpenProt. In that case, Module 2 outputs can be filtered to include only altProts from OpenProt, which significantly shortens the process. This option is not integrated into MosaicProt because OpenProt focuses only on a few species. However, matching FASTA-formatted Model 2 outputs to FASTA-formatted OpenProt entries by BLASTP is a straightforward task manageable by a helper script provided in the MosaicProt GitHub repository. As an alternative, Module 3 can model chimeric proteins based on all theoretically possible altProts regardless of the evidence for their conservation or translation. This flexibility is important for two reasons. Firstly, most translated altORFs are not conserved. Secondly, most MS-validated chimeric peptides in our dataset were modeled with altORFs that have no MS evidence. Thus, using altProt inputs with zero-support for translation may be an informative approach alternative to our procedure, provided such altProts are fed into Module 3 in sequential groups, not as one large block, as was explained in our data article [2]. If a user wishes to model chimeric peptides with altProts that are likely to be translated, like we did in our study, the refProt-free output sequences from Module 2 can be processed with the software for BLASTP analysis (e.g., DIAMOND, [37]) and searching for MS peptide matches (e.g., SearchGUI, [35]; PeptideShaker, [36]). This intermediate step will generate two inputs for Module 3 that can be handled either independently or as a combined list. In our study, we modelled chimeric proteins separately based on conserved altProts and MS-validated altProts. The models are generated according to three scenarios, which depend on the location of an altORF relative to its refORF and/or other altORFs (Fig. 2). In the first scenario, an altORF is completely embedded within its refORF or another long altORF, without extending into untranslated regions (UTRs). In the second scenario, an altORF can partially overlap with its refORF so that part of the altORF is located in a UTR. In the third scenario, there is no overlap between an altORF and a refORF (or between two altORFs) so that the altORF is fully located within a UTR. Scenario 3 addresses an unconventional situation that may require the forward slippage of ribosomes by more than two nucleotides. It was included in our study [2] to test whether the translation products of non-overlapping ORFs can be combined in a single continuous polypeptide by frameshifting over longer distances (up to ten nucleotides). This means two ORFs joined in this fashion could belong either to different reading frames or to the same reading frame, in contrast to the other two scenarios. An example of the modeling for the first scenario is shown in Supplementary Figures S1 and S2. Below, we describe each of the three modules.

### 2.1 The ORF detector module

The first module identifies and *in silico* translates all ORFs found in a given transcriptome. The module was designed to make no discrimination between refORFs and altORFs. This is an important feature because the status of an ORF as a refORF or an altORF can change over time [38], [39]. In the context of research on PRF using our approach, ORFs are best defined as sufficiently long regions of transcript sequences that are free of in-frame stop codons, regardless of the presence of the canonical start codon AUG. These can be segments of variable length (a user-defined parameter, see Results) found either between two in-frame stop codons [38] or between a stop codon and either end of a transcript. The module assigns each generated amino acid sequence a unique tag (identifier) containing information about the genomic locus, reading frame, ORF coordinates on the source transcript, and the ORF length on the transcript. This detailed tagging process is essential for downstream analyses as it provides clear and accessible information about each ORF, enabling efficient filtering, categorization, and subsequent modeling steps. For example, tag MtrunA17_Chr6g0457461_3F_438–596_159 reads as follows: the ORF is in a spliced annotated transcript of locus MtrunA17_Chr6g0457461, forward frame 3, which starts at nucleotide 438 and ends at nucleotide 596, with the total length of 159 nucleotides. Users can provide their FASTA-formatted nucleotide sequences (for example, an entire transcriptome) and specify a minimum length threshold for identifying ORFs. By default, the threshold is set to 30 aa (the setting of OpenProt), but users can customize it according to their needs. For example, in our study on chimeric peptides in *M. truncatula*, we used the threshold of 20 aa to enable the analysis of many altORFs. The module generates a single FASTA file with refProt and altProt sequences combined. Each FASTA-formatted sequence is supplied with a tag described above.

### 2.2 The altProt/refProt separator module

The second module is dedicated to filtering out refORF products, or refProts, from the output generated by the first module. It produces two FASTA-formatted files: the altProt file and the refProt file, both of which serve as inputs for Module 3 (the chimeric modeler module). AltProt and refProt sequences in these files have MosaicProt-specific unique identifiers described above, which are essential for modeling chimeric peptides. A user defines which ORFs are considered as refORFs by providing a FASTA-formatted canonical proteome list (amino acid sequences downloadable from a genome browser of an organism under study). After filtering out refProts, the FASTA file with altProts produced by the module can be used in at least four different ways. Firstly, it can serve as a search database for matching MS peptides found in biological samples, which can provide the most direct evidence for translation of non-canonical amino acid sequences. We employed SearchGUI v. 4.0.41 [35] and its partner tool PeptideShaker v. 2.0.33 [36] for MS searches. Secondly, the altProt list can be subjected to the global protein sequence similarity search using BLASTP with one of the well-annotated reference protein databases. We used UniProt [40] as the database and DIAMOND as a search tool [37]. Such analysis can deliver evidence for conservation of altORFs at the protein level regardless of the evidence for their translation. Thirdly, the list can be matched with FASTA-formatted altProts downloadable from OpenProt using our helper script for BLASTP analysis (the GitHub page of MosaicProt). The output of this option is a list of altProts with MosaicProt identifiers, which can be used as input for Module 3 (the chimeric modeler module). The fourth possible use of the Module 2 outputs is to feed them directly to the third module, if the goal is to model chimeric proteins with all possible altORFs above a given length in the entire transcriptome or in a subset of it. The altProt/refProt separator module, therefore, is a critical step in the pipeline, as it refines the dataset to focus on alternative protein-coding sequences. By producing a comprehensive list of altProts, Modules 1 and 2 enable subsequent analyses that can reveal altORFs’ translational activity, conservation, and potential role in the proteome regardless of their involvement in PRF.

### 2.3 The chimeric modeler module

This module uses four input files to generate chimeric protein models: (1) a transcriptome file, (2) a file that contains altProts, (3) a file that contains refProts, and (4) a text file (extension “.txt”) that contains a user-defined list of altProt identifiers (not altProt sequences). File 1 is downloadable from a genome browser of a species under study. Alternatively, it can be generated *de novo* from a transcriptomic study on an organism with no genome annotation. It must be FASTA-formatted. The purpose of File 1 is to provide MosaicProt with a map to find overlapping refORFs and altORFs defined by the remaining three files. It should be noted that scenarios that involve altORFs overlap with each other regardless of their overlap with a refORF are also considered. FASTA-formatted Files 2 and 3, which contain altProts and refProts, respectively, are generated by the second module. The txt-formatted fourth file that contains a user-defined list of altProt identifiers tells MosaicProt which altORFs to consider in modeling. It may correspond to conserved altProts, MS-supported altProts, or both. In our study, Çakır et al. [2], the evidence for conservation and MS-validation was obtained with the aid of DIAMOND [37] and SearchGUI+PeptideShaker [35], [36], respectively, which are external tools not integrated in MosaicProt. The txt-formatted fourth file may also include MosaicProt-specific identifiers of altProts with Ribo-Seq evidence for translation (for example, altProts listed in OpenProt). Thus, the fourth file may contain a subset of altProt identifiers from the second file. In case users wish to conduct the modeling based on ORFs regardless of their conservation and/or translation status, the fourth file may either contain altProt identifiers from the entire second file or identifiers of altProts selected based on criteria other than conservation, MS support, or Ribo-Seq evidence, for example, altProts that originate from one specific chromosome. Whereas altORFs are treated differently in our analysis depending on evidence for their translation and conservation [2], MosaicProt does not intrinsically discriminate between translated, non-translated, conserved, and non-conserved altORFs. It simply focuses on ORFs that the user wishes to include in modeling, based on any criteria (File 4). It does not even make any conceptual discrimination between altORFs and refORFs. Instead, it relies upon the user’s definition of altORFs and refORFs (Files 2 and 3, respectively). Nevertheless, MosaicProt assumes that user-designated refORFs are translated. This assumption is necessary but not entirely accurate, since most annotated refProts have never been validated with MS proteomics or other methods. As a result, chimeric peptides MS-validated with the aid of MosaicProt do not only reveal the existence of previously unknown PRF sites but also serve as the first MS-based evidence for translation of corresponding refORFs, if their chimeric models were based on altORF/refORF pairs. This is a significant, although not obvious, added bonus of our method. The chimeric modeler module combines three functions: searching, modeling, and filtering. First, it finds overlapping ORFs predefined by input File 4. Then, for each overlapping pair of ORFs, it generates a set of chimeric protein models starting at specific positions around the beginning of the overlapping region. The exact starting positions and other parameters are specific to each scenario (Fig. 2). The modeling proceeds from left to right in one-nucleotide steps, called iterations, each corresponding to a putative PRF position. PRF values −2, −1, + 1, and + 2 are considered in our study. The signs correspond to the direction of the frameshift and the number indicates the length of the frameshift in nucleotides. For example, a + 2 frameshift advances the ribosome in the forward direction by two nucleotides relative to its normal progression. Our algorithm also addresses special cases where two ORFs do not overlap but are separated by one to ten nucleotides. Translation products of such ORFs can potentially be joined by forward frameshifts with PRF values up to + 10. Models generated under each scenario contain two short amino acid sequences from two reading frames, the left arm and the right arm, joined at a putative PRF site. When the algorithm occasionally produces a model with an empty position that corresponds to an incorporated stop codon, such defective models are removed automatically so that the output file contains only continuous sequences. The final output of the chimeric modeler module is a set of chimeric protein sequences that adhere to the specific PRF scenarios and parameters defined in the study. These models represent theoretical constructs that can be matched with MS data to identify PRF-derived chimeric peptides.

Each model generated by Module 3 is supplied with a unique identifier/tag that is based on identifiers of corresponding ORFs *in silico* translated by Module 1. For example, chimeric peptide CP98 (RuBisCo) was MS-validated using the model with the following identifier: MtrunA17_Chr6g0457461_3F_438–596_159_MtrunA17_Chr6g0457461_2F_170–715_546_+ 2_iteration_6_Within_3prime_of_altORF. This long tag combines tags of two ORFs, the shorter one (159 nt) being an altORF and the longer one (715 nt) being a refORF. The term “+ 2” indicates the PRF value of the corresponding frameshifting event while the term “iteration_6” shows that this model corresponds to the sixth one-nucleotide step from the starting point. Which ORF is shown first, and which one is shown second depends on technical aspects of the overlapping region and does not convey any biological information. “Within_3prime_of_altORF” is a tag that reflects the position of an altORF relative to the refORF and the side at which the models are simulated. This specific tag indicates that an altORF is nested within the refORF and that models are simulated in the 3′ portion of the altORF (right side). The prime symbol (') is fully spelled to avoid computational problems. Supplementary Data S1 contains comprehensive description and examples of eight possible tags generated by MosaicProt.

### 2.4 Benchmarking MosaicProt

The runtime performance of MosaicProt was benchmarked separately for each module using annotated transcriptomes of six representative eukaryotic species, which are shown here in the alphabetical order: *Arabidopsis thaliana*, *Caenorhabditis elegans*, *Danio rerio*, *Gallus gallus*, *M. truncatula*, and *Homo sapiens*. These transcriptomes were downloaded from Ensembl and EnsemblPlants as FASTA files: *A. thaliana* (TAIR10), *C. elegans* (WBcel235), *D. rerio* (GRCz11), *G. gallus* (GRCg7b), *H. sapiens* (GRCh38), and *M. truncatula* (MtrunA17r5.0_ANR). The transcriptomes differ in parameters such as the number and average length of transcripts (Supplementary Table S1 and Supplementary Figures S3-S5). They also have different numbers of altORFs and refORFs (see benchmarking of Module 2 below). All benchmarking analyses were performed using MosaicProt version 0.1.13. The first two modules, detect_ORFs (Module 1) and separate_ORFs (Module 2), were executed using a single thread. Parallel scalability was assessed only for simulate_chimeric_proteins (Module 3), which supports multithreading. Benchmarking was executed on a high-performance computing cluster. Wall-clock runtime was recorded for each run. Benchmarking results were then compared with transcriptome parameters to evaluate the influence of transcriptome size, transcript length, ORF number, and parallelization on performance. The results of this analysis are summarized in Supplementary Figures S6-S10.

*H. sapiens* exhibits the largest and most complex transcriptome, comprising approximately 330,000 transcripts with a mean length of 2259 nt and a median length of 1824 nt, reflecting extensive alternative splicing and gene diversity typical of mammalian genomes. In contrast, *M. truncatula* has the shortest mean transcript length (1504 nt) and a median length of 1276 nt, followed by *C. elegans* with a mean of 1624 nt and a median of 1221 nt, consistent with their compact transcriptome architectures. The two plant species, *A. thaliana* and *M. truncatula*, generally have shorter transcripts compared to animals (Supplementary Table S1). Among vertebrates, *G. gallus* exhibits the longest mean and median transcript lengths (mean: 3400 nt; median: 2866 nt), while *D. rerio* displays intermediate values (mean: 2110 nt; median: 1504 nt), reflecting moderate transcriptome complexity (Supplementary Table S1 and Supplementary Figures S3–S5).

The smallest transcriptome of *C. elegans* was consistently processed at the fastest rate by all three modules. The human transcriptome, which is the largest, was processed at the slowest rate. However, there was module-specific variation in the runtime performance of the remaining four transcriptomes. The runtimes are outlined below in the ascending order.

Module 1: *M. truncatula* → *A. thaliana* → *G. gallus* → *D. rerio*

Module 2: *M. truncatula → A. thaliana* → *D. rerio* → *G. gallus*

Module 3: *M. truncatula → A. thaliana → D. rerio* → G. *gallus* (one thread)

Module 3: *A. thaliana* → *M. truncatula* → *D. rerio* → *G. gallus* (multiple threads)

Here, we attempt to analyze the sources of this variation. *G. gallus* has fewer transcripts than *D. rerio*. Thus, *G. gallus* was processed faster than *D. rerio* by Module 1. However, transcripts of *G. gallus* have a larger cumulative length. Consequently, Modules 2 and 3 needed more time to process transcripts of *G. gallus*. This indicates that Module 1, which identifies ORFs, is rather insensitive to the average and cumulative lengths of transcripts. Its runtime depends mostly on the number of transcripts (Supplementary Figure S7). With the increased number of threads, *A. thaliana* was processed faster than *M. truncatula* by Module 3 (Supplementary Figure S10) even though all transcriptome parameters of *A. thaliana* are larger, including the number of ORFs (Supplementary Table S1). This difference can be explained by the higher number of chimeric models generated for *M. truncatula* (25,712) compared to *A. thaliana* (20,931). The larger number of models increases computational workload and thus runtime, outweighing the modest differences in transcriptome size between the two species. The number of generated chimeric models depends on specific features of the sampled altORFs such as positions and frequencies of stop codons, which define the cumulative overlap length and the number of resulting models.

Now, let us consider the details of the runtime performance of each module. The total number of transcripts and, to a lesser extent, the cumulative transcriptome length were the best predictors of the Module 1 runtime, indicating that the ORF detection process scales linearly with data volume. Species with smaller and more compact transcriptomes required approximately a minute, whereas larger and more complex transcriptomes, like *H. sapiens*, were processed by Module 1 within several minutes (Supplementary Figures S6 and S7).

Module 2 of MosaicProt, which separates refORFs and altORFs, showed performance patterns consistent with the transcriptome size (Supplementary Figure S8 and Supplementary Table S1). Runtime scaled proportionally with the number of detected ORFs and the total output size, demonstrating predictable computational scaling across species. Smaller transcriptomes such as *C. elegans* and *M. truncatula* were completed within 10–15 min, while intermediate-sized transcriptomes (*A. thaliana*, *D. rerio*, and *G. gallus*) required 20–40 min. As expected, the human transcriptome showed the highest computational demand, requiring approximately 1100 minutes due to its large transcript count, cumulative size, and extensive number of detected alternative ORFs (>5 million).

For benchmarking of Module 3, responsible for chimeric protein modeling, a standardized candidate ORF list was generated from the altProts identified by Module 2. From each species’ altProt file, all ORF headers were extracted, and every 1,000th entry was selected to ensure an even sampling across the dataset. A total of 200 representative altORFs per species were used to simulate chimeric proteins. Module 3 was benchmarked to assess parallel scalability across species and processor counts. Runtime decreased sharply with the number of processors, demonstrating efficient parallelization (Supplementary Figure S10).

For all species, runtime dropped rapidly between 1 and 20 threads, with diminishing gains beyond approximately 20 threads. *H. sapiens* exhibited the highest computational demand due to its large transcriptome size and complex ORF set but still achieved a near 10-fold reduction in runtime when using multiple threads. Processing smaller transcriptomes such as *C. elegans* and *A. thaliana* required only a few minutes, even at low thread counts, while *M. truncatula, G. gallus*, and *D. rerio* showed intermediate scaling performance. At higher thread counts (>50), runtime slightly increased, likely due to overhead (the additional resources like processing time and memory) from thread management and limited candidate distribution (one or a few) per thread, reflecting the expected trade-off in fine-grained parallelization.

It is important to mention the limitations of these benchmarking results. Runtime measurements were obtained under a specific hardware and software configuration, and actual performance may vary depending on the system architecture, memory bandwidth, and parallelization overhead. In addition, candidate chimeric models in Module 3 were generated from a fixed subset of 200 entries per species to enable cross-species comparison, which may not fully capture runtime behavior under real-scale datasets.

The benchmarking was carried out using the Scientific Compute Cluster of the Society for Scientific Data Processing (GWDG), the joint data center of Max Planck Society for the Advancement of Science (MPG) and the University of Göttingen. The node is equipped with two AMD EPYC Milan processors (32 cores per socket, 128 CPU cores total) and 1 TB of system memory.

The code of the benchmarking pipeline, together with example files, is available at https://github.com/umutcakir/chimsource.

## 3 Theory/calculation

### 3.1 General principles

In this section, we exemplify the calculation of the number of chimeric protein models generated per switch from one ORF to another. It is meant to reflect the computational challenge of including all possible chimeric protein models in the analysis. For the left side of the first modeling scenario (Fig. 2, Supplementary Figures S1 and S2), the number (N) of modeled chimeric proteins that correspond to the shift from the refORF (yellow) to the altORF (light red) can be calculated according to Equation 1:

$$N\;=\;\left(\mathrm{overlap length in aa}-\mathrm{minimum size in aa}+\;1\right)*\;2$$

The factor “2” accounts for the two possible directions of switching between frames (backward and forward PRF events). Thus, 42 chimeric proteins are modeled for the shift from the refORF to the altORF if the altORF is 90 nt long (30 aa) and if the minimum size of the altORF or the refORF part considered in the modeled chimeric protein is chosen to be 10 aa. In this example, the overlap length corresponds to the whole length of the embedded altORF in amino acids. To illustrate the scale of this process, let us consider a dataset containing 10,000 altORFs, each measuring 90 nt in length and embedded within their respective refORFs. The total number of chimeric models generated for such a dataset can be calculated as follows: 10,000 × 42 for the left side plus 10,000 × 42 for the right side, which makes 840,000 chimeric models. Since many altORFs in our study are longer than 90 nt, the actual number of models can be much larger for every increment in the altORF length. This explains why it is practical to consider only a subset of possible models in one MS-based study.

In the first scenario illustrated in Supplementary Figure S1, chimeric proteins can be modeled in two ways that correspond to frameshifts either from the refORF to the altORF or the other way around. In Supplementary Figure S2, chimeric proteins modeled for a frameshift from the refORF to the altORF are shown. Here, if the refORF and the altORF are located in the third frame and the first frame, respectively, the ribosome can switch from the third frame to the first frame in two different ways: via + 1 and −2 frameshifting events. There are 42 possible chimeric proteins that can theoretically correspond to the frameshift from the refORF to the altORF if the minimum size of a sequence contributed by either ORF in a chimeric model is chosen to be 10 aa. First, the modeling algorithm takes the region between the nucleotides 390 (10 aa upstream of the altORF start) and 510 (30 aa downstream of the altORF start). Then it proceeds in one-nucleotide steps (iterations) from left to right, moving the frameshift position with each step until the last model contains 30 aa from the refORF and 10 aa from the altORF. For this example, there are 21 iterations and 21 modeled chimeric proteins. The same principle, with some variations, was applied for other scenarios (Fig. 2). For the left side of Scenario 1, the iteration process begins 10 aa upstream of the altORF starting point, whereas for the right side, it starts 30 aa upstream of the altORF end. The first model on the right side includes 10 aa from the altORF and 30 aa from the refORF, while the last model contains 30 aa from the altORF and 10 aa from the refORF. The minimum contribution of 10 aa from the first ORF in overlapping pairs is a consistent setting for both sides of Scenario 1. However, in Scenario 2, the last model may include only 1 aa from the second ORF, as this setting accommodates cases such as the copper-related protein in *E. coli*, where a frameshift product included a single amino acid from the alternative frame [9]. In Scenario 3, the altORF and refORF do not overlap, but their separation does not exceed 10 nt. Frameshifting events in this scenario involve forward slippage, allowing translation products of non-overlapping ORFs to join. For this scenario, the minimum size of a sequence contributed by each ORF in a chimeric model was set to 20 aa, which means only frameshifting events that join two non-overlapping ORFs were considered. The overall length of each chimeric protein fragment was limited to only 40 aa, a parameter common for all the three scenarios. These settings were chosen based on the length range typical for MS-derived peptides (7–35 aa, [41]). The idea was to make sure an MS peptide spans the frameshift position rather than matches either part of the chimeric model. This, however, does not mean we anticipate the chimeric products to be of that short length only. As we hypothesize, they can be long molecules, but we need to focus on the short fragments corresponding to the frameshift sites in order to validate their chimeric nature.

Below we describe further details of each scenario, corresponding settings, and the calculations. The basis for the chimeric protein modeling algorithm is visualized in Fig. 2. Position k corresponds to the beginning of the refORF. Positions l and m correspond to the beginning and the end of altORF number 1, respectively. Position n corresponds to the beginning of altORF number 3. The algorithm focuses on a representative subset of all possible situations. For example, starting the iteration process at the end of altORF number 2, the end of refORF, or the middle of altORF number 1 was not considered, which helped alleviate the search database inflation problem [42], [43]. Because Module 3 has a scalable design, users can adapt it to capture an even greater diversity of PRF events. The current version of MosaicProt serves as a proof of concept, and we encourage the research community to develop it further for the maximal coverage of diverse scenarios. However, at least some of them will be included in the next release of MosaicProt, which is discussed at the end of this manuscript.

### 3.2 Scenario 1

For the altORFs denoted with number 1 in Fig. 2, chimeric proteins are modeled separately at the 5’- and 3’-portions of the altORFs. At the 5’-end of the altORF, the algorithm starts modeling 10 aa upstream of position l and proceeds until position l + 30 aa in 21 iterations. All corresponding chimeric models in this region are generated: the first chimeric protein model is composed of 10 aa from the refORF and 30 aa from the altORF, and the last one is composed of 30 aa from the refORF and 10 aa from the altORF. In contrast, at the 3’-side of the altORF, which is used for the modeling of the switch to the refORF, 30 aa upstream of position m is taken and extended to position m + 10 aa in 21 iterations, so that the last iteration stops at the end of the altORF. All chimeric models in this region are generated: the first model is composed of 10 aa from the altProt and 30 aa from the refProt, and the last model is composed of 30 aa from the altProt and 10 aa from the refProt. The sequence of the first 10 aa is the same for all 21 models in this case. Likewise, the last 10 aa of all 21 models are shared.

### 3.3 Scenario 2

When an altORF that starts in the 5’-UTR overlaps with its refORF (number 2 in Fig. 2), chimeric proteins are modeled in the following way: 10 aa upstream of position k are taken and extended via iterations to position k + 30 aa. All chimeric protein models in this region are generated. The first chimeric protein model (the first iteration) is composed of 10 aa from the altProt and 30 aa from the refProt. However, the last chimeric model (iteration 30) is composed of 39 aa from the altProt and just 1 aa from the refProt. All generated chimeric models share the first 10 aa. Their number is calculated as follows: 30 × 2 = 60, where “30” indicates the number of iterations from one to 30, and “2” refers to the separate consideration of backward and forward frameshifting events. When an altORF that ends in the 3’-UTR overlaps with its refORF (number 3 in Fig. 2), chimeric proteins are modeled in the following way: 10 aa upstream of position n is taken and extended via iterations to position n + 30 aa. All chimeric models in this region are generated. The first model is composed of 10 aa from the refProt and 30 aa from the altProt. The last model is composed of 39 aa from the refProt and one amino acid from the altProt. Similar to the 5’-UTR side of this scenario, all generated chimeric models share the first 10 aa. Likewise, their number is 30 × 2 = 60.

### 3.4 Scenario 3

When an altORF does not overlap with its refORF, but the gap between the altORF and the refORF is equal to or less than 10 nt (altORFs 4 and 5 in Fig. 2), chimeric proteins are modeled in the following way. If an altORF is located at the 5’-UTR (4), the last 20 aa of the altProt and the first 20 aa of the refProt are joined in sequential order. Likewise, if an altORF is located at the 3’-UTR (5), the last 20 aa of the refORF and the first 20 aa of the altProt are joined in sequential order. The number of modeled chimeric proteins is limited to one for each pair of ORFs.

### 3.5 Other scenarios

In addition to the scenarios mentioned above, an altORF may span the whole refORF; that is, the beginning and the end of the altORF can be located at the 5’-UTR and the 3’-UTR, respectively (altORF 6 in Fig. 2). In this case, the shifts from altORF 6 to the refORF (the 5’-side) and from the refORF to altORF 6 (the 3’-side) are considered as special cases of Scenario 2, with a few modifications. Namely, 10 aa upstream of position k is taken and extended to position k + 30. While the first chimeric model is composed of 10 aa from the altProt and 30 aa from the refProt, the last chimeric model is composed of 39 aa from the altProt and one amino acid from the refProt. Likewise, the altORF end is handled almost as in the case with altORF 3 with the difference that 30 aa is taken upstream of the refORF stop codon and extended to the total length of 40 aa (30 iterations). The first chimeric model is generated as 10 aa from the refProt plus 30 aa from the altProt. The last chimeric model is composed of 39 aa from the refProt and one amino acid from the altProt. In rare cases, chimeric proteins cannot be modeled as explained above. For instance, if an altORF is shorter than 90 nt and is embedded in its refORF, or if there is no 10 aa long sequence upstream of position k, the length of chimeric protein models can be less than 40 aa.

Here we explained in detail the overlapping of an altORF with its refORF. However, an altORF can overlap with another altORF. In such cases, one altORF was considered as a refORF, and many possible chimeric models were generated with the same procedure. Furthermore, if more than two ORFs overlap, all paired combinations of ORFs were modeled, and possible chimeric models were generated with the same procedure. For instance, if two altORFs (A and B) and one refORF (C) overlap in the same transcript, the following chimeric models were generated: A & C, B & C, A & B. Here, the ampersand symbol (“&”) denotes the frameshift event that combines products of two overlapping ORFs into a chimeric protein model. By considering all possible pairs, the algorithm ensures that every biologically plausible chimeric protein is captured, reflecting the full complexity of overlapping ORFs in multi-ORF transcripts.

## 4 Results

In our study, we set the minimal length of altORFs to 60 nt, which is a reasonable threshold compatible with the BLASTP analysis. Queries shorter than 20 aa are unlikely to be processed accurately by BLASTP, as follows from one of the original articles on BLASTP [44], technical documentation of BLAST [45], and one empirical study [46]. For comparison, OpenProt uses a threshold of 90 nt [25], [23], [24]. The rationale of using a lower threshold was to maximize the number of altORFs. The effect of dropping this setting from 30 to 20 was as expected: many more altORFs were available for analysis. However, this does not compromise the false discovery rate (FDR), which was kept below 1 % in our study, because the altORFs were processed in chunks as a part of the two-step search for MS peptides (see below; also, see our data article [2]). The sequential operation of the ORF detector module and the altProt/refProt separator module identified 875,356 altORFs in the annotated transcriptome v. 5.1.7, which was downloaded from the *M. truncatula* genome portal MtrunA17r5.0-ANR [47]. The transcriptome files that served as inputs for the first module contained transcripts of four types: mRNA, ncRNA, rRNA, and tRNA; 51,317 non-microRNA transcripts in total. Although a precursor of miRNA, pre-miRNA, was shown to act as a template for translation [48], mature miRNA sequences are too short for the identification of ORFs longer than 59 nt. Thus, they were excluded from the analysis. Excessively large numbers of amino acid sequences included in the search database for the identification of corresponding MS peptides are associated with the phenomenon of database inflation [42], [43]. To avoid this problem, in our study, we limited the modeling of chimeric proteins to altORFs that are either MS-supported (805) or conserved (13,078) or MS-supported and conserved at the same time (103); 13,780 unique sequences in total. Different criteria can be used to define what a conserved altORF is. We used a simplified definition of “conserved” by focusing on altORF products that have at least one match to a subject in the UniProt database with a percent identity value of at least 70 and an e-value at most 0.001 in DIAMOND search [37].

Using 51,317 transcript sequences and 13,780 conserved and/or MS-supported altORFs as inputs, the chimeric modeler module generated 473,871 unique models of chimeric proteins, out of which 472,087 models were built from overlapping ORFs. The remaining 1784 models were built from non-overlapping ORFs that are not more than 10 nt apart. The lengths of the arms and the overall length of each model were chosen so that the MS peptides cover the PRF sites. This design maximizes the efficiency of detection because the typical length of MS peptides in proteomic studies is 7–35 aa [41]. Accordingly, most models are close to 40 aa in length. Among models that were supported with MS proteomics in our study, the length varied between 28 and 41 aa. The length of each arm varied between one and 40 aa. Typically, 42 models were generated per hypothetical shift from one ORF to another, half of which corresponded to backward hypothetical PRF sites (shift by one or two nucleotides from right to left) and the other half contained forward hypothetical PRF sites (shift by one or two nucleotides from left to right). Models generated with such parameters were used as a search database for matching MS peptides present in 16 biological samples generated by three studies [49], [50], [51]. Corresponding MS proteomic data with identifiers PXD002692, PXD013606, and PXD022278, respectively, are fully available to the research community. They were downloaded from ProteomeXchange [52], [53]. The MS searches were conducted independently for models based on conserved and MS-supported altORFs. Likewise, the MS proteomic data from each of the three studies were searched independently. Furthermore, while a regular MS search procedure was applied to non-mRNA-based models and models that come from MS-supported altORFs, a two-step search procedure was used for mRNA-based models that come from conserved altORFs [54]. The same principle was applied during the MS-validation of non-chimeric altProts that were used for the modeling of chimeric events where conserved and mRNA-derived altProts outnumber MS-supported and non-mRNA derived altProts. The purpose of this two-step approach was to alleviate the database inflation effect, which otherwise is expected to increase the rate of false negatives and to compromise the confidence of detection when the search database is large [42], [43]. Details of our two-step procedure based on the target-decoy strategy are summarized in the data article associated with this pipeline (Supplementary Tables S10 and S11 in [2]). In total, 156 chimeric peptides were supported with MS proteomic data, all of which came from overlapping ORFs. None of 1784 models generated from non-overlapping ORFs received MS support in our study, which is not an artifact of the method because there was no intrinsic bias against such models. This indicates that PRF activity joining products of non-overlapping ORFs in one chimeric sequence probably does not exist in *M. truncatula*, at least under our criteria (shifts up to 10 nt). This phenomenon, termed programmed translational bypassing/jumping, is well-documented in bacteriophages [55] and yeast mitochondria [56], [57]. Typically, it involves the bypassing of sequences much longer than 10 nt. Out of 156 MS-supported models, the majority came from conserved altORFs (135). Remarkably, although no rRNA and tRNA-based altORFs were supported with MS (the MS search that preceded the modeling), six chimeric models from rRNA transcripts and one from a tRNA transcript had matching MS peptides. Eleven MS-supported chimeric models corresponded to ncRNA transcripts. The remaining 138 models came from mRNA transcripts. Peptides for the majority of MS-supported models (129) were found in just one sample each, and the remaining 27 models had peptides found simultaneously in two to nine MS samples. Although approximately equal numbers of chimeric models were generated per each PRF value (-2, −1, +1, and +2), sequences that correspond to backward PRF events were significantly overrepresented among MS-supported models. Statistical analysis conducted on these data revealed 37 significant observations and 14 significant associations among various parameters of MS-supported models, their peptides, and corresponding transcripts, which suggests the biological relevance of the dataset [2].

## 5 Discussion

Although the approach described here was proposed for the *de novo* detection of diverse PRF events in our earlier work [19], to the best of our knowledge, it has not been applied by other groups so far. Presumably, the approach was perceived as too computationally intensive and too prone to false discoveries. In our recent study, we demonstrated that, although the approach requires considerable computational effort, it enabled the discovery of 156 candidates for translated chimeric peptides, some of which correspond to multiple PRF events per transcript (two to three per transcript; 15 non-repeat loci and eight repeat loci). Careful analysis of such multi-PRF sequences revealed two likely candidates for mosaic proteins, which correspond to a putative protein-synthesizing GTPase (a homolog of elongation-factor 1-alpha) and a putative ribulose-bisphosphate carboxylase (RuBisCo). These two completely unrelated loci share the same pattern and parameters of PRF events, which supports the biological significance of these shared characteristics [2]. No candidate mosaic sequence is known so far beyond viruses. Thus, our new pipeline generated data that deserve dedicated functional studies, with the potential for major discoveries, including the experimental validation of the mosaic translation hypothesis [19] and evidence for translation of chimeric peptides from non-mRNA transcripts.

The pipeline presented here is applicable to any organism, providing a tool to identify diverse PRF events without requiring prior knowledge of PRF sequences. This makes our approach uniquely suited to discovering novel PRF sites and events that might evade detection by conventional methods. The pipeline can identify PRF events that join the products of non-overlapping ORFs. With some modifications, the same principle can be applied to detect frameshifts “longer” than two nucleotides. In viruses, “long” frameshifts with the slippage of the ribosome by up to six nucleotides have been known for a long time [58], [59]. Although we focused on PRF events with four values (-2, −1, +1, and +2) and have not identified any chimeric peptides that come from non-overlapping ORFs, the sequence diversity of putative PRF sites is enormous. They are almost completely unique to each MS peptide. This emphasizes that our approach can be far more efficient in the search for novel PRF sequences compared to other methods, which can reveal new determinants and mechanisms of PRF in any organism.

As a technical note, modeling chimeric proteins with our pipeline using all ORFs present in a transcriptome (Fig. 1 Option 2) would create a database of an astronomic size, which would be difficult to use efficiently for the MS proteomic searches. This is why we limited modeling to conserved and MS-validated altORFs only. However, it may be informative to use altORFs for modeling chimeric proteins regardless of their conservation and/or translation status if the MS search is based on products of only a fraction of the genome, for example, one chromosome or only one chromosome arm. Such an approach would not cause database inflation and could identify PRF events that have so far evaded detection. It is known that many translated altProts have either no or very low similarity to annotated proteins ([21]; Orr at al., 2020). This is also evident from the fact that, out of 805 MS-supported non-chimeric altORFs detected in our study, only 103 are conserved. Moreover, the conservation of most altORFs is limited to the *M. truncatula* genome or genomes of other legume plants. Thus, PRF events that involve taxonomically restricted ORFs can be a promising subject for future studies using our pipeline.

In the next release of MosaicProt, we plan to enhance flexibility and broaden functionality of the pipeline. Fixed settings such as the length of chimeric models (currently 40) and the minimal number of amino acids contributed from each ORF (currently 10) will be made adjustable by users. We also plan to introduce the second mode of operation of Module 3 (the chimeric modeler module), which will be available as an alternative to the current mode. At present, Module 3 generates a range of models where PRF sites are scattered between two positions. This operation mode results in most models being asymmetric with regard to the length of segments to the left and to the right from the PRF site. We call this mode of operation the asymmetric or scattered mode. It can capture unusual PRF events of Scenario 2 such as described in bacteria, where only one amino acid is incorporated into the chimeric protein from the alternative frame [9]. However, this mode can miss some PRF events, depending on the availability of enzymatic cleavage sites. To address this technical limitation, we plan to incorporate the second mode of operation that will always center the PRF site in the model. This operation mode will result in all models being symmetric with regard to the length of segments to the left and to the right from the PRF site. Consequently, it will be called the symmetric or centered mode. It will skip models that are too close to stop codons but will permit the detection of PRF sites missed by the asymmetric mode. Another planned improvement concerns broadening the diversity of scenarios such as starting the iteration process at the end of altORF number 2, the end of the refORF, or the middle of altORF number 1 (Fig. 2).

## 6 Conclusions

The method and the code described here offer the first approach for the *de novo* identification of PRF events, enabling the systematic and unbiased exploration of frameshifting. It operates independently of prior knowledge about PRF sequences or predefined annotations, facilitating the discovery of both conventional and unconventional PRF events. After our pilot study in *M. truncatula*, we hope that the application of this pipeline in a wide range of organisms will enrich the knowledge about the true diversity of proteomes. Importantly, this knowledge can be the basis for the discovery of mechanisms and phenomena that have remained unnoticed so far.

## Author statement

All authors have read and approved the submission of this revised manuscript to Computational and Structural Biotechnology Journal. The manuscript is not under consideration by any other journal.

## CRediT authorship contribution statement

**Kryvoruchko Igor S:** Writing – review & editing, Writing – original draft, Visualization, Validation, Supervision, Resources, Project administration, Methodology, Investigation, Funding acquisition, Formal analysis, Data curation, Conceptualization. **Noujoud Gabed:** Writing – review & editing, Validation, Resources, Investigation, Formal analysis, Conceptualization. **Ali Yurtseven:** Writing – review & editing, Software, Methodology, Formal analysis. **Umut Çakır:** Writing – review & editing, Writing – original draft, Visualization, Validation, Software, Resources, Methodology, Investigation, Funding acquisition, Formal analysis, Data curation, Conceptualization.

## Declaration of Competing Interest

The authors declare no competing interest relevant to this study.

## Data Availability

The code for this pipeline, along with instructions on installation and functionality, is accessible from the following GitHub repository and PyPI project: https://github.com/umutcakir/mosaicprot https://pypi.org/project/mosaicprot For convenience, input and output file examples are also provided. The helper script for BLASTP-based matching Module 2 output sequences to OpenProt entries (Option 4 in Fig. 1) is provided in the same GitHub repository.
