# Supplementary material for: A universal pipeline MosaicProt enables large-scale modeling and detection of chimeric protein sequences for studies on programmed ribosomal frameshifting
Source: Comput Struct Biotechnol J. 2025 Nov 12;27:5105–15. doi: 10.1016/j.csbj.2025.11.023 (PMC12664050; doi:10.1016/j.csbj.2025.11.023)
Supplement: Supplementary file 2 — Supplementary material [file mmc2.pdf]

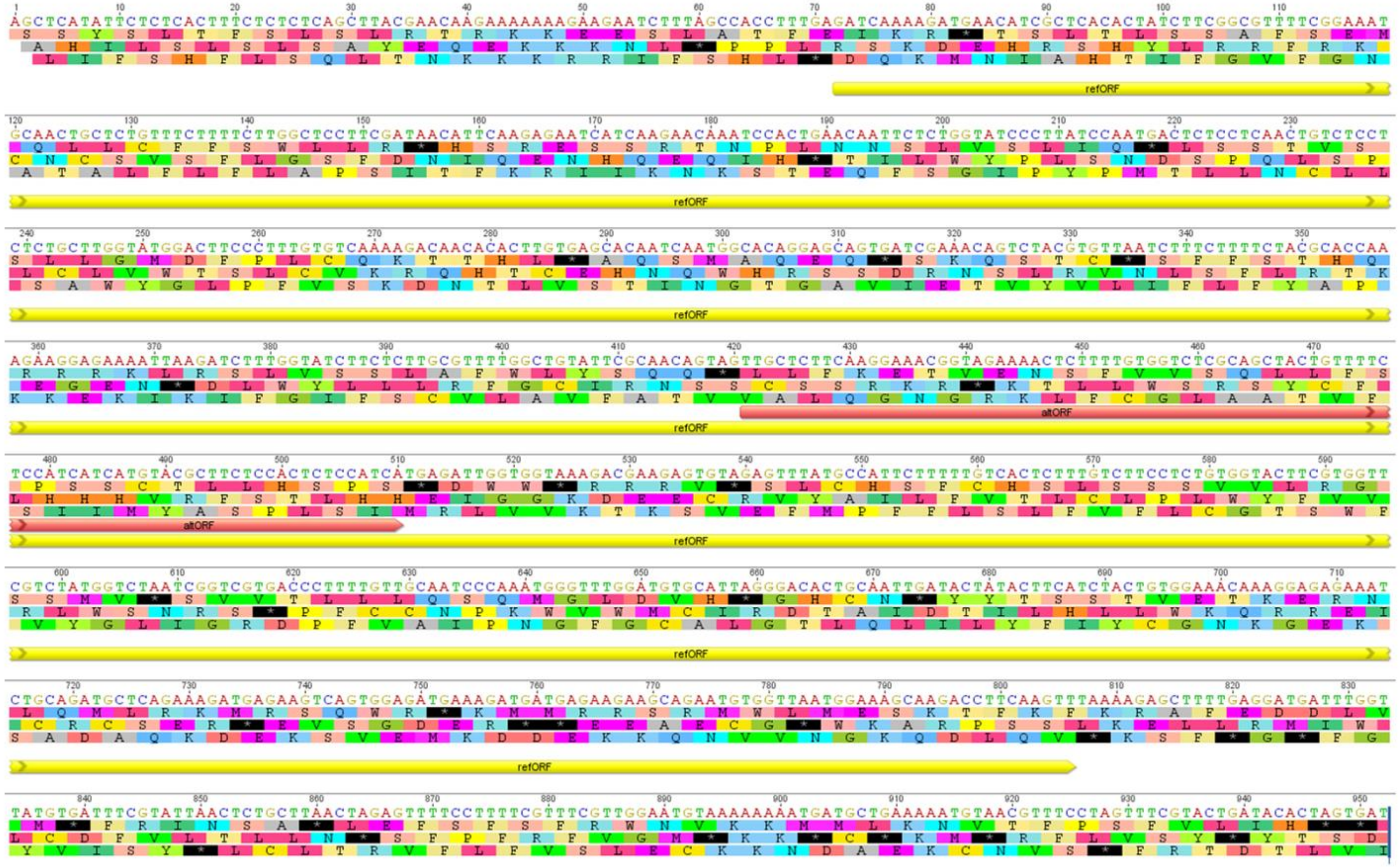

Supplementary Figure S1. Generation of model sequences for frameshifted proteins translated around a candidate programmed ribosomal frameshifting (PRF) site is a computational challenge. This image illustrates a hypothetical transcript and its three-frame translation. There is one reference open reading frame (refORF, yellow) in frame 3 and one translated alternative open reading frame (altORF, light red) in frame 1 that overlaps the refORF. The exact location of the hypothetical PRF event is unknown.

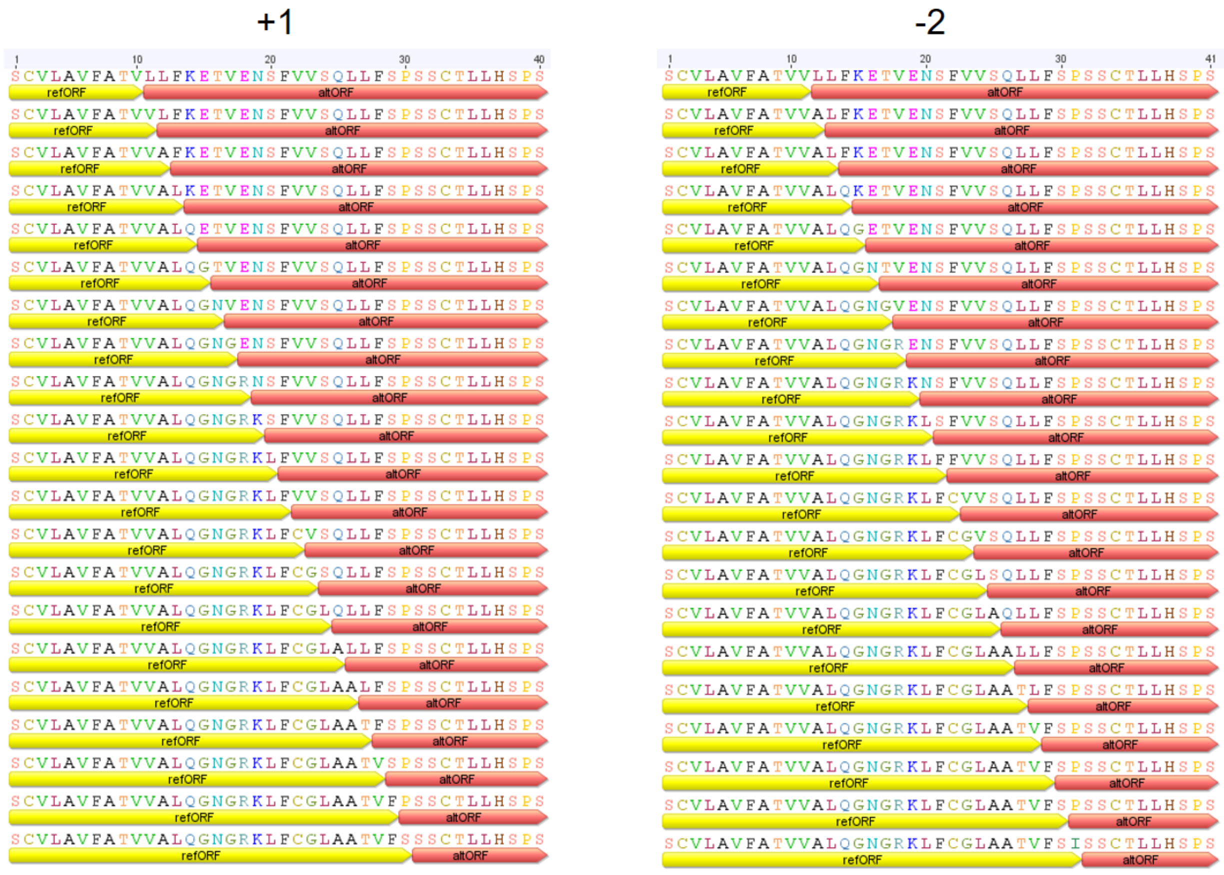

Supplementary Figure S2. A modeling example for the left-hand side of Supplementary Figure S1. There are 42 chimeric proteins that can theoretically correspond to the shift from the refORF to the altORF of a certain length (30 aa in this example). “42” comes from the following calculation: (30 - 10 + 1) x 2, or more generally (overlap length - 10 + 1) x 2. “10” is the minimal size of an altORF product in a chimeric protein (an arbitrarily set threshold convenient for the reliable matching to MS peptides on either side of the shift). The left column depicts chimeric proteins (partial view, only the hypothetical PRF site shown) resulting from skipping one nucleotide (PRF value +1). The right column lists proteins resulting from a backward shift by two nucleotides (PRF value -2).

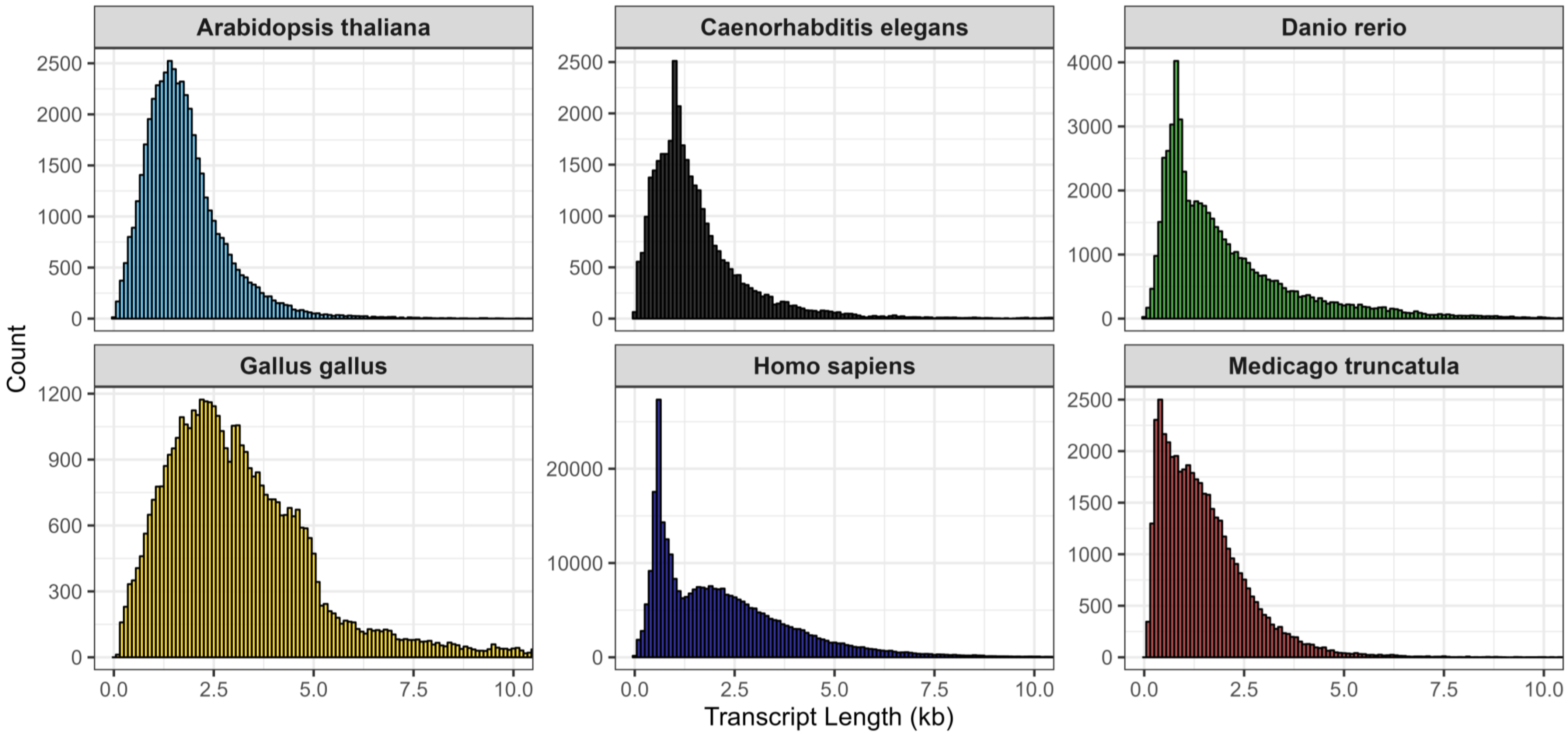

Supplementary Figure S3. Transcript length distributions of six representative eukaryotic species used for benchmarking of MosaicProt. The graphs exclude transcripts longer than 10 kilobase pairs (kb). The bin width in all histograms is 100 nucleotides. Note the very large discrepancy in counts (*Homo sapiens* vs other species).

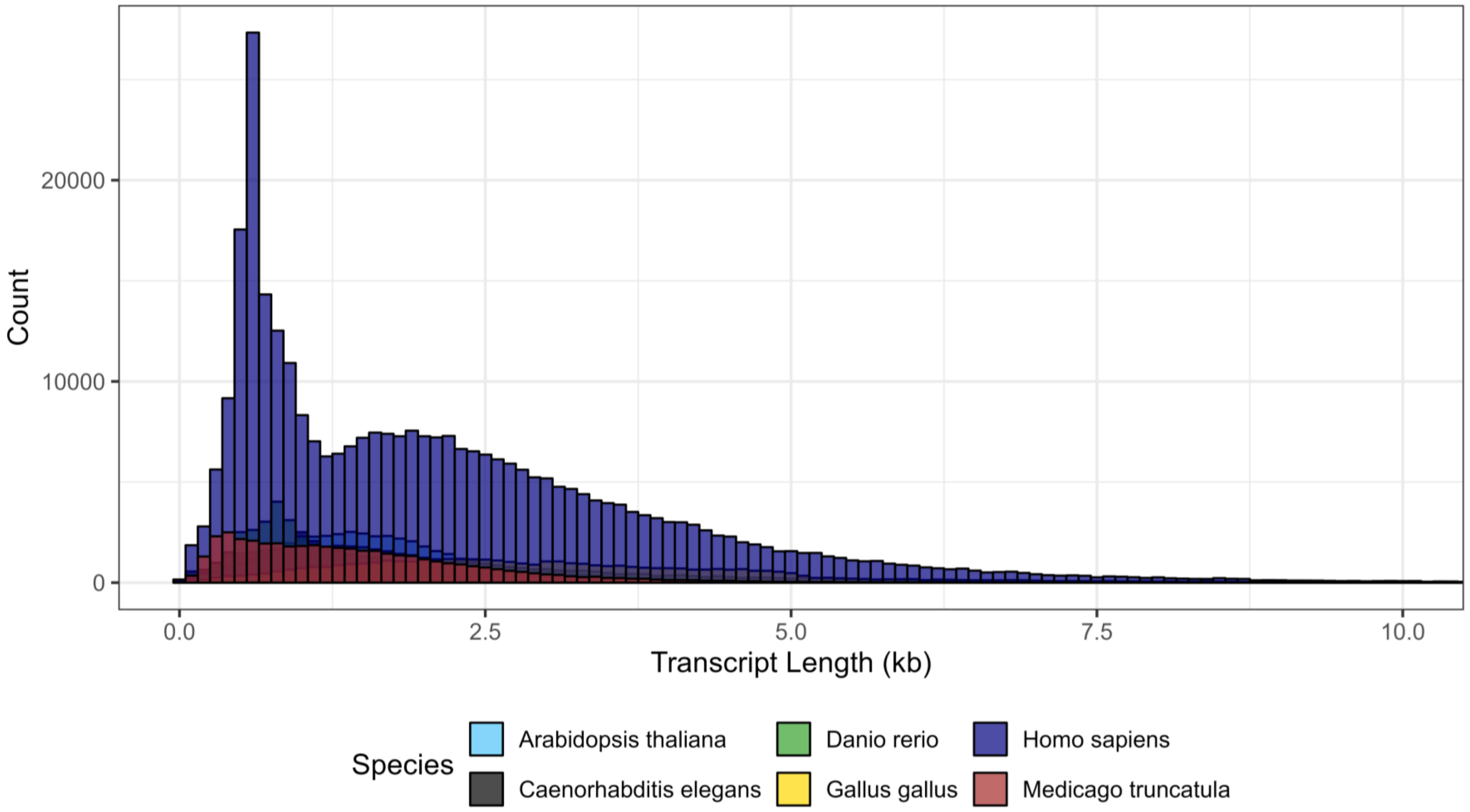

Supplementary Figure S4. An overlay image of transcript length distributions of six species used for benchmarking of MosaicProt. The graphs exclude transcripts longer than 10 kilobase pairs (kb). The bin width in all histograms is 100 nucleotides. Note the very large discrepancy in counts (*Homo sapiens* vs other species).

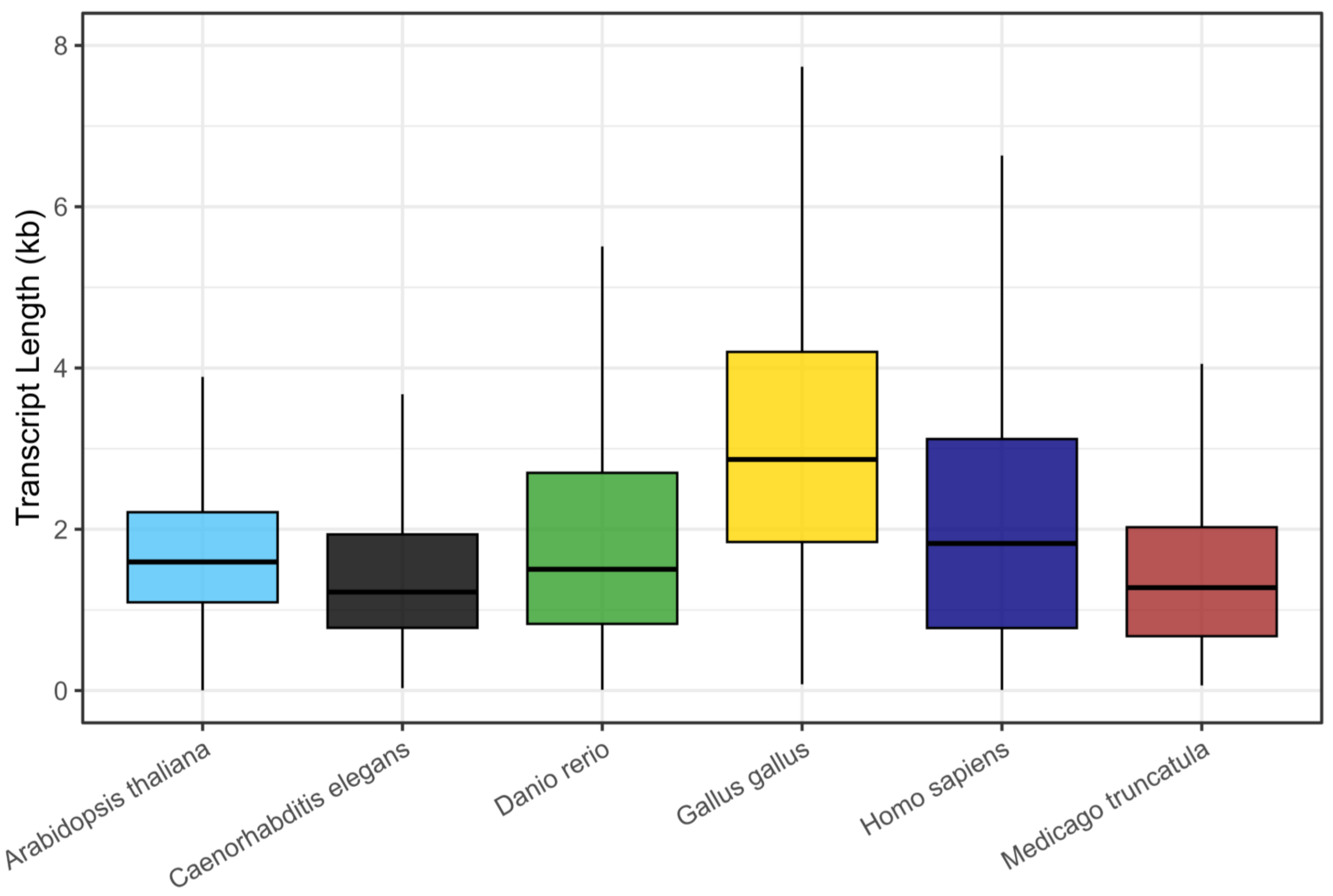

Supplementary Figure S5. Boxplots of transcript length distributions of six species used for benchmarking of MosaicProt. Outliers are not shown. Note that the human transcripts are not the longest ones.

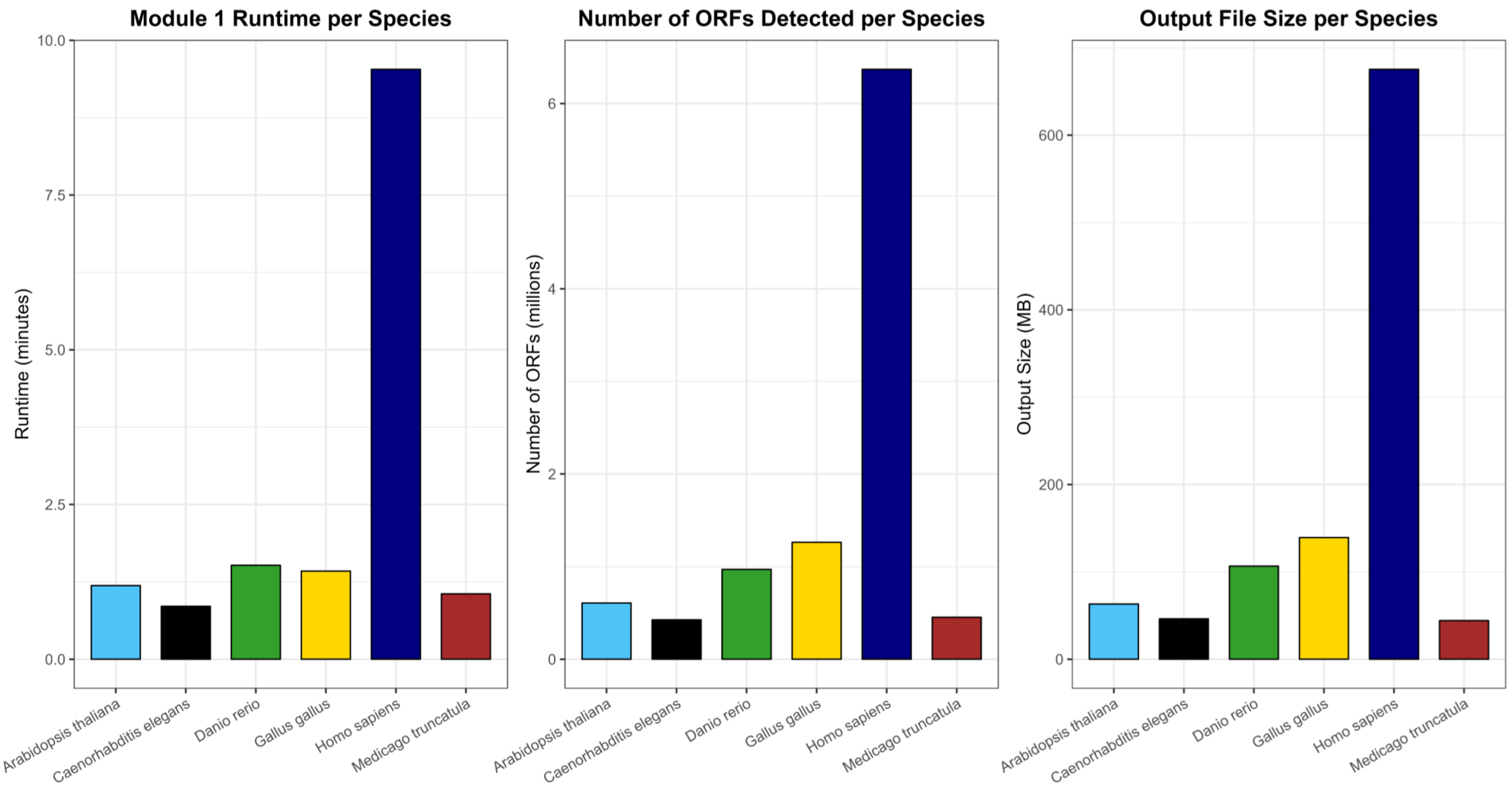

Supplementary Figure S6. Benchmarking (runtime) results for Module 1, the ORF detector module (command “detect_ORFs”). The transcriptome with the largest number of transcripts (*Homo sapiens*, Supplementary Table S1) stands out in all three categories: the runtime, the ORF number, and the file size. “MB” stands for megabytes.

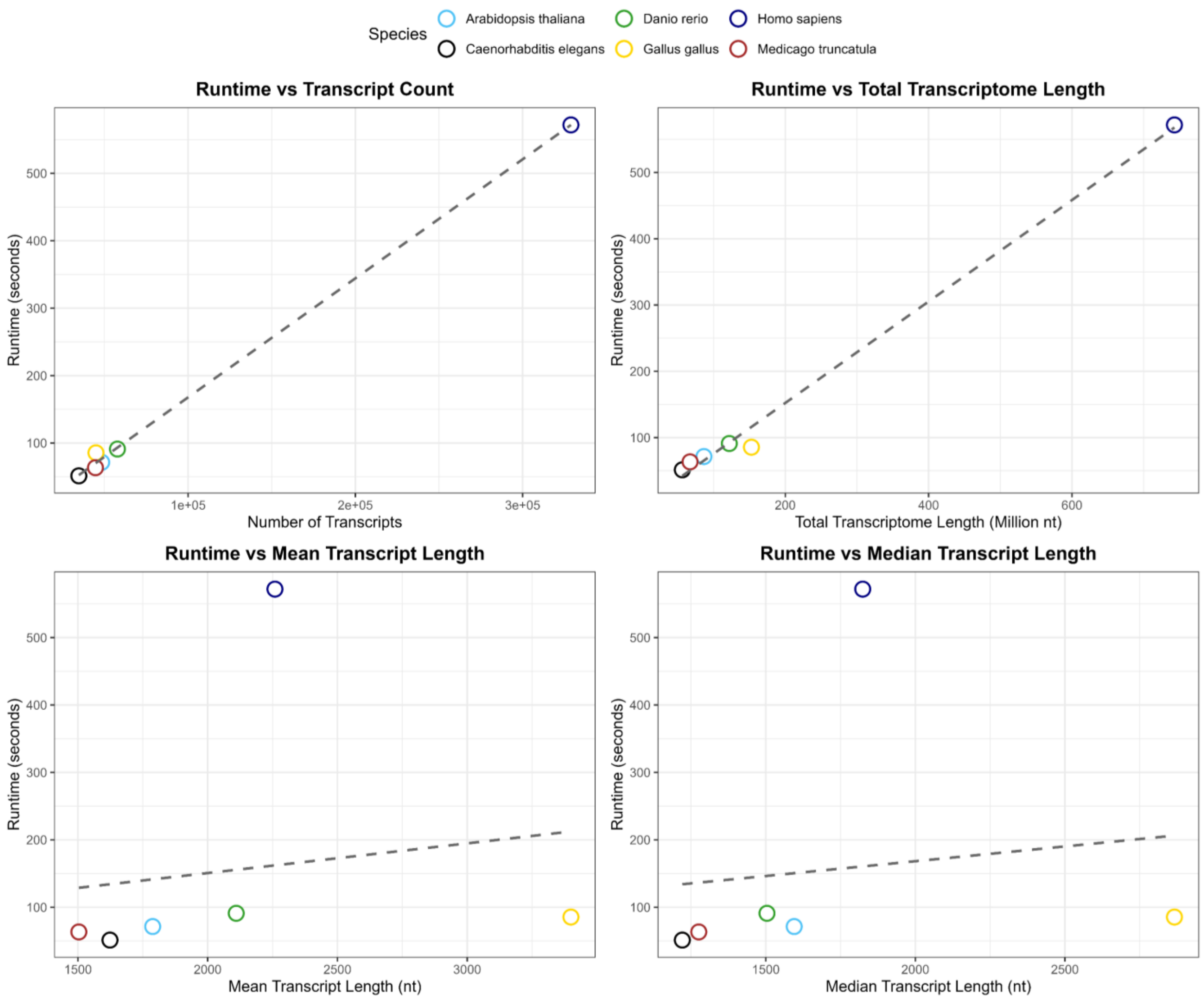

Supplementary Figure S7. Module 1 runtime correlation with transcriptome features. The runtime increases with the transcript count and transcriptome length but does not depend much on the average transcript length, as can be seen from the positions of *Homo sapiens* data points on these graphs. The human transcriptome has a very large number and total length of transcripts (Supplementary Table S1); thus, the runtime is much longer compared with other transcriptomes. “nt” stands for nucleotides.

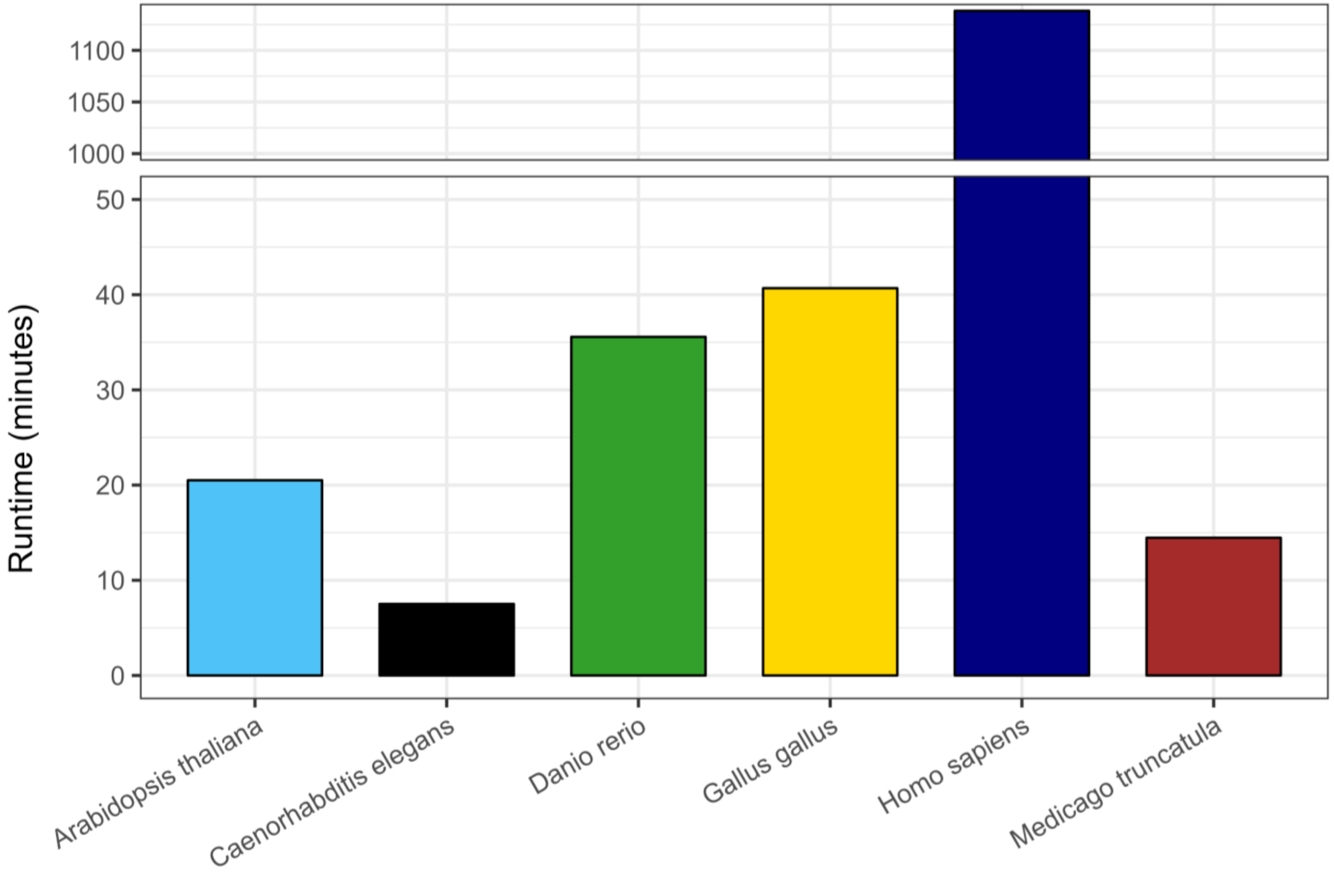

Supplementary Figure S8. Benchmarking (runtime) results for Module 2, the altProt/refProt separator module (command "separate_ORFs"). Note the nearly 20-fold difference between the time required for *Homo sapiens* and for other species. This is due to the very large number and cumulative length of transcripts in the human transcriptome (Supplementary Table S1).

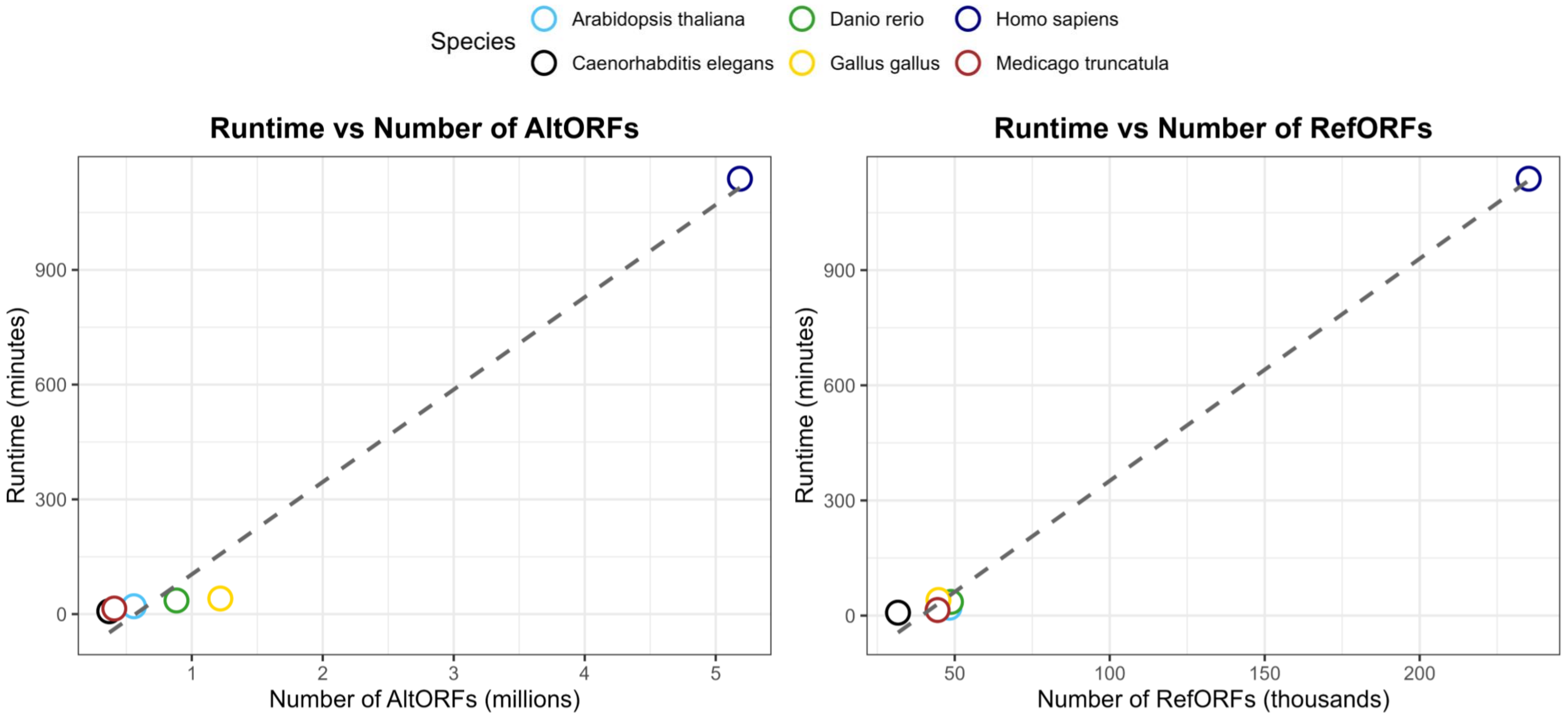

Supplementary Figure S9. Module 2 runtime correlation with the number of altORFs and refORFs. The runtime and the number of ORFs increase with the transcript count and cumulative transcriptome length (Supplementary Table S1), as can be seen from the positions of *Homo sapiens* data points on these graphs. For smaller genomes, the runtime does not correlate with the number of ORFs. However, if two transcriptomes are identical with regard to the count of transcripts and their cumulative length, a transcriptome with the smaller number of ORFs will be processed slightly faster because of less writing to the disk.

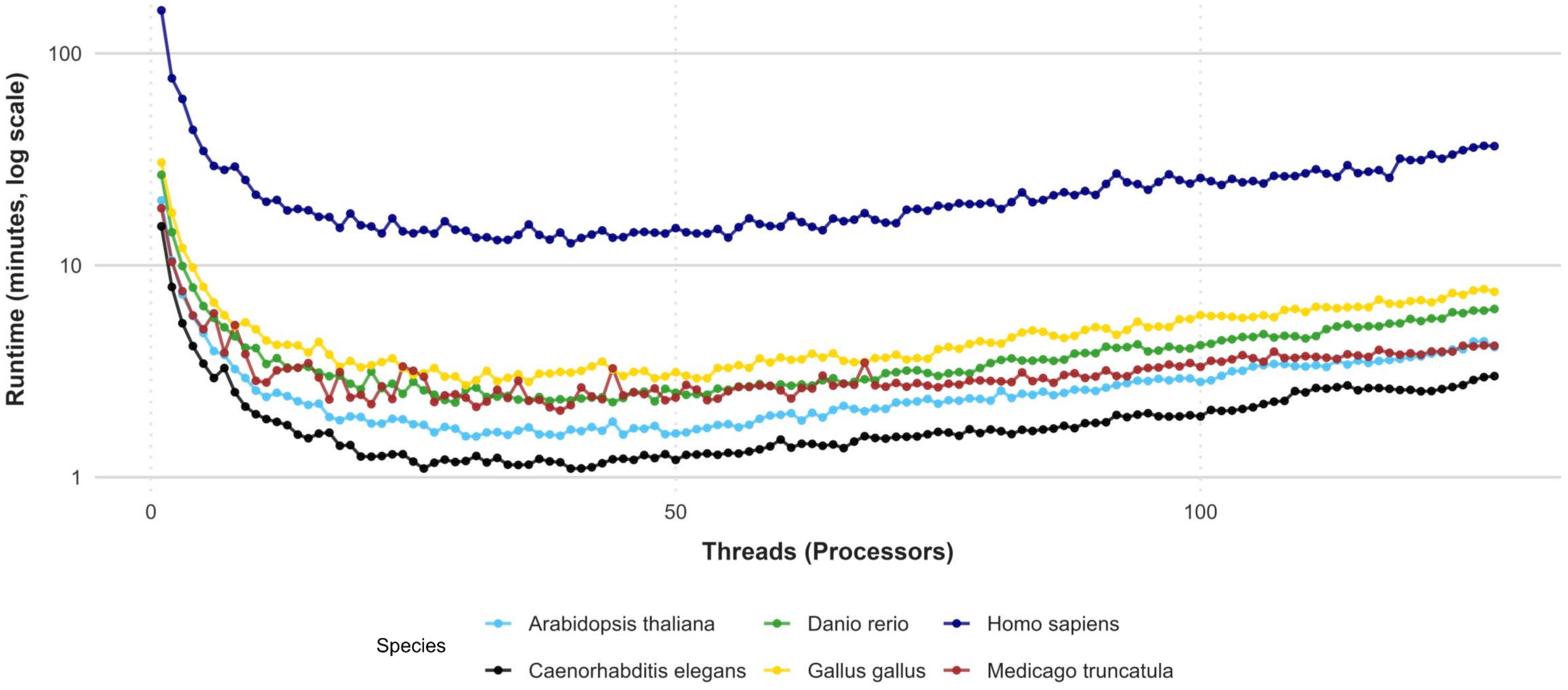

Supplementary Figure S10. Benchmarking (runtime) results for Module 3, the chimeric modeler module (command "simulate_chimeric_proteins"). Note that using more than 20 processors does not improve the runtime regardless of transcriptome parameters.
