# Supplementary material for: A universal pipeline MosaicProt enables large-scale modeling and detection of chimeric protein sequences for studies on programmed ribosomal frameshifting
Source: Comput Struct Biotechnol J. 2025 Nov 12;27:5105–15. doi: 10.1016/j.csbj.2025.11.023 (PMC12664050; doi:10.1016/j.csbj.2025.11.023)
Supplement: Supplementary file 3 — Supplementary material [file mmc3.pdf]

Supplementary Table S1. Descriptive statistics of six representative eukaryotic transcriptomes used for benchmarking of MosaicProt

Species	Number of transcripts	Total length, nt	Mean length, nt	Median length, nt	Number of models*
Arabidopsis thaliana	48,359	86,469,099	1,788	1,595	20,931
Caenorhabditis elegans	34,689	56,317,021	1,624	1,221	24,912
Danio rerio	57,775	121,902,979	2,110	1,504	18,656
Gallus gallus	44,937	152,771,019	3,400	2,866	15,710
Homo sapiens	328,868	742,967,561	2,259	1,824	15,445
Medicago truncatula	44,642	67,116,870	1,504	1,276	25,712

“nt” stands for nucleotides.
*This is a parameter of a sample of 200 altORFs, not a parameter of the entire transcriptome.
